# Follicular fluid-derived small extracellular vesicles during individual *in vitro* maturation improve blastocyst development

**DOI:** 10.3389/fvets.2025.1644542

**Published:** 2025-09-18

**Authors:** Nima Azari-Dolatabad, Davoud Eshghi, Camilla Benedetti, Andrea Fernandez Montoro, Lei Xie, Fabiola Le Graffric Molto, Sarah E. Moorey, An Hendrix, Geert Opsomer, Jo L. M. R. Leroy, Ann Van Soom, Krishna Chaitanya Pavani, Osvaldo Bogado Pascottini

**Affiliations:** ^1^Department of Internal Medicine, Reproduction and Population Medicine, Faculty of Veterinary Medicine, Ghent University, Ghent, Belgium; ^2^Department of Veterinary Sciences, Gamete Research Center, Veterinary Physiology and Biochemistry, University of Antwerp, Antwerp, Belgium; ^3^Department of Animal Science, University of Tennessee, Knoxville, TN, United States; ^4^Department of Clinical Sciences, Faculty of Veterinary Medicine, Ferdowsi University of Mashhad, Mashhad, Iran; ^5^Department of Animal Sciences, Biotechnology and Reproduction of Farm Animals, University of Göttingen, Göttingen, Germany; ^6^Department for Reproductive Medicine, Ghent University Hospital, Ghent, Belgium; ^7^School of Veterinary Medicine, University College Dublin, Dublin, Ireland

**Keywords:** assisted reproduction, follicle, blastocyst, small vesicles, exosomes

## Abstract

**Introduction:**

We evaluated the impact of follicular fluid-derived small extracellular vesicles (FF-sEVs) supplementation during oocyte maturation in vitro on bovine embryo outcomes, comparing group and individual culture systems.

**Methods:**

Follicular fluid was aspirated from dominant follicles of four nulliparous Holstein heifers at 4.5 days post-ovulation. Small extracellular vesicles were isolated, characterized, and pooled to ensure balanced donor contribution. To confirm uptake, FF-sEVs were fluorescently labelled and co-cultured with cumulus-oocyte complexes (COCs) during *in vitro* maturation. Fluorescent labelling confirmed FF-sEVs internalization by oocytes and granulosa cells. Next, COCs were matured *in vitro* with FF-sEVs at varying concentrations (group system: 0, 5, 10, 25, 50 μg/mL; individual system: 0, 6.5, 12.5, 25 μg/mL), fertilized, and cultured. Blastocyst quality was assessed via differential-apoptotic staining.

**Results:**

In group culture, the control group exhibited higher day 8 blastocyst rates compared to 10, 25, and 50 μg/mL FF-sEVs groups, while 5 μg/mL FF-sEVs showed no difference. Blastocysts developed from oocytes matured in 25 and 50 μg/mL groups had reduced total cell numbers versus controls and groups matured in lower FF-sEVs concentrations. Conversely, individual maturation with 6.5 μg/mL FF-sEVs enhanced day 8 blastocyst rate, total cell counts, inner cell mass, and reduced apoptotic ratios compared to all other groups.

**Discussion and conclusion:**

We propose that intercellular communication in group cultures, potentially mediated by endogenous embryotropins (including sEVs), may mask FF-sEVs benefits. In individual systems, where such interactions are absent (or minimal), FF-sEVs significantly improved embryo competence. These findings underscore FF-sEVs as a promising tool to refine assisted reproductive technologies, contingent on culture conditions.

## Introduction

In assisted reproductive technologies (ART), both embryo production and quality are critical to enhancing pregnancy success rates. As researchers and clinicians explore innovative strategies to enhance *in vitro* embryo production (IVP), extracellular vesicles (EVs) have emerged as key players in reproductive biology (1–8). Extracellular vesicles are a diverse group of membrane-bound particles, including exosomes, microvesicles, apoptotic bodies, and ectosomes, which vary in size, origin, and surface markers (9–11). Found in nearly all biological fluids, EVs carry a cargo of bioactive molecules (e.g., proteins, lipids, and nucleic acids) that are delivered to recipient cells, influencing cellular functions (12–14). These properties make EVs essential regulators of key reproductive processes.

The follicular environment, rich in proteins, lipids, nucleic acids, and hormones, plays a vital role in supporting oocyte maturation. Furthermore, the follicular fluid (FF) has been identified as a significant source of EVs (15–17). These FF-EVs are thought to mediate oocyte maturation and early embryo development by transferring bioactive molecules to target cells (18–20). However, their effects may vary depending on the stage of follicular growth. For instance, while EVs from preovulatory FF have shown some positive effects, those from small follicles appear to enhance cumulus cell expansion and embryo development more effectively than EVs from larger follicles (21, 22). This suggests that the functional properties of EVs are influenced by the follicular development stage (23), highlighting the need for further research to optimize their use in ART.

*In vitro* supplementation of FF-derived small EVs (sEVs) has been shown to promote a plethora of events in cumulus oocyte complexes (COCs) (20, 22, 24), however, these findings are based on group culture production systems (25). In group culture, oocyte maturation, fertilization, and embryo development are conducted with at least 25 oocytes or embryos, typically at a density of 1 zygote per 2 μL of culture medium. Under serum-free conditions, this method yields blastocyst rates of up to 40–50% by day 8 post-insemination (26). In contrast, individually cultured embryos, even at a density of 1:20 (with a minimum droplet volume of 20 μL), show reduced blastocyst rates (30–40%) and greater variability, sometimes dropping below 30% when maturation and fertilization are also performed individually (27, 28). Individually cultured blastocysts also exhibit lower hatching rates, reduced total cell numbers, and higher apoptotic cell ratios compared to group-cultured embryos (29, 30). These differences are attributed to the absence of autocrine and paracrine signaling from neighboring oocytes or embryos in individual culture systems (29). Despite these challenges, developing a reliable single-oocyte culture system is essential, as it enables individual monitoring of embryo viability and quality, which is critical for optimizing ART outcomes (30).

To date, no studies have investigated the effects of supplementing FF-sEVs into the maturation medium in a fully individual culture system. Additionally, the dose-specific impact of FF-sEVs supplementation in a complete serum-free IVP process under group culture conditions remains unclear. We hypothesized that adding FF-sEVs to the *in vitro* maturation (IVM) medium could improve embryo development and quality in both group and individual culture systems. The objective of this study was to evaluate the effects of supplementing FF-sEVs at varying concentrations in group and individual culture under serum-free conditions, with the aim of assessing their influence on subsequent embryo development and quality.

## Materials and methods

### Media and reagents

Tissue culture medium (TCM)-199, gentamycin, and phosphate-buffered saline (PBS) were purchased from Life Technologies Europe (Ghent, Belgium). All other chemicals, unless otherwise specified, were obtained from Sigma-Aldrich (Overijse, Belgium). Prior to use, all media were filtered using a 0.22 μm filter (GE Healthcare-Whatman, Diegem, Belgium).

### Adherence to MISEV2023 guidelines

Follicular fluid-sEVs collection, pre-processing, storage, isolation, characterization, internalization assay, and functional analysis were performed in accordance with the Minimal Information for Studies of Extracellular Vesicles 2023 guidelines (31).

### Follicular fluid collection

All animal procedures were approved by the Ethical Committee of Animal Testing of the University of Antwerp (Antwerp, Belgium) and the Flemish Government (ECD-dossier 2019–44). Four healthy Holstein heifers (12–14 months old) from a single herd were selected, with cyclicity confirmed via transrectal ultrasonography based on the presence of a corpus luteum. To synchronize oestrus for FF collection, a double Ovsynch protocol was administered: heifers received 0.02 mg GnRH (Receptal®, MSD Animal Health) on day −26, 500 μg cloprostenol (Estrumate®, MSD Animal Health) on day −19, and a second GnRH dose on day −17. The protocol was repeated 7 days later (GnRH on day −10, cloprostenol on day −3, and GnRH on day −1), with ovulation designated as day 0.

On day 4.5 post-ovulation, the dominant follicle (~8 mm diameter) of the first follicular wave was aspirated via transvaginal ultrasonography. Briefly, heifers were restrained, and the perineum was cleaned with iodide soap, ethanol (70%), and dried. After epidural anaesthesia (2 mL of 4% Procain HCl, VMD, Belgium), a 7.5-MHz ultrasound transducer (Pie Medical Imaging, Netherlands) guided follicle puncture. Follicular fluid was aspirated using a 5 mL syringe connected via a stainless-steel and silicone line. Blood-free FF samples were transferred to microcentrifuge tubes, transported at 4°C within 1 h, centrifuged (10,000 × g, 10 min, 4°C), filtered (0.22 μm, GE Healthcare-Whatman, Belgium), and stored at −80°C.

### Isolation and characterization of small extracellular vesicles

Small EVs were isolated from FF using an OptiPrep™ density gradient ultracentrifugation (ODG UC) protocol adapted from Van Deun et al. (32) and by Asaadi et al. (16) for bovine FF samples. Briefly, iodixanol gradients (5, 10, 20, and 40%) were prepared by mixing OptiPrep™ (60% w/v iodixanol) with homogenization buffer (10 mM Tris–HCl, 1 mM EDTA, 0.25 M sucrose, pH 7.4). Gradients were layered in a 16.8 mL polyallomer tube (Beckman Coulter, Brea, CA, USA) as follows: 4 mL 40%, 4 mL 20%, 4 mL 10%, and 3.5 mL 5% iodixanol solutions. One mL of FF was overlaid onto the gradient and centrifuged at 100,000 × g for 18 h at 4°C (SW 32.1 Ti rotor, Beckman Coulter). Small-EVs were collected from fractions 8–10 of 16 gradient layers, pooled, diluted in 13 mL PBS, and centrifuged (100,000 × g, 3 h, 4°C). The pellet was resuspended in 100 μL PBS and stored at −80°C for downstream analysis.

Small EVs characterization included transmission electron microscopy (TEM), nanoparticle tracking analysis (NTA), and Western blotting, following validated protocols established by previous publications (28, 33). For TEM, thawed FF-sEVs were adsorbed onto Formvar/carbon-coated copper grids (Aurion, Leiden, Netherlands), stained with 1% uranyl acetate, and imaged using a JEM 1400 Plus microscope (JEOL, Benelux). Nanoparticle tracking analysis was performed using a NanoSight LM10 (Malvern Instruments, UK): FF-sEVs were diluted in PBS (3 × 10^8^–1 × 10^9^ particles/mL), injected into the chamber, and analyzed with NTA Software 3.2 (three 60 s videos per sample; detection threshold 3, camera level 13). For Western blotting, FF-sEVs lysates were denatured in reducing buffer (0.005% bromophenol blue, 3% 2-mercaptoethanol, 9.2% SDS, 40% glycerol, 0.5 M Tris–HCl, pH 6.8) at 95°C for 5 min, separated by SDS-PAGE, and transferred to nitrocellulose membranes (Bio-Rad, Hercules, CA, USA). Membranes were blocked with 5% bovine serum albumin (BSA)/0.5% Tween-20, incubated overnight at 4°C with primary antibodies against CD63 (Abcam ab68418, 1:250), TSG101 (Santa Cruz sc-7964, 1:1000), and CD9 (CST-D3H4P, 1:1000), washed with 0.5% Tween-20/PBS, AGO-2 (Abcam ab32381, 1:1000), and incubated with HRP-conjugated anti-mouse (1:3000) or anti-rabbit (1:4000) IgG secondary antibodies (GE Healthcare). Chemiluminescence (Western Bright Sirius, Advansta) was detected using a Proxima 2,850 imager (IsoGen Life Sciences, Netherlands).

### Assessment of small extracellular vesicles intake during *in vitro* maturation

Follicular fluid-sEVs were labelled using the ExoGlow-Protein EV Labelling Kit (System Biosciences, Palo Alto, CA) according to the manufacturer’s instructions, and as previously described for FF-sEVs in the equine species (34). Briefly, FF-sEVs were incubated with a 1:500 dilution of the 500X fluorescent labelling dye in PBS for 20 min at 37°C under gentle agitation. Unbound dye was removed by adding ExoQuick-TC solution, incubating for 4 h at 4°C, and centrifuging at 10,000 × g for 10 min. The supernatant was discarded, and the FF-sEVs pellet was resuspended in 100 μL of filtered PBS and kept on ice.

For maturation, labelled FF-sEVs were added to 500 μL of pre-equilibrated maturation medium (38.5°C, 5% CO₂) containing 60 COCs. A negative control was prepared by incubating PBS with labelling dye (omitting FF-sEVs), following the same protocol, and adding the solution to maturation medium with 60 COCs. After 22 h of IVM (38.5°C, 5% CO₂), COCs were fixated in 4% paraformaldehyde at 4°C overnight. Samples were stained with 0.1% Hoechst 33342 (pan-nuclear fluorescent dye) for 10 min, mounted on glass slides (≤10 COCs per slide) in DABCO-based antifade mounting medium, and cover slipped. Imaging was performed within the same day using a confocal microscope (Leica Microsystems GmbH, Wetzlar, Germany). Image overlays were generated using ImageJ software v1.52d (Wayne Rasband, National Institutes of Health, USA).

### Experimental design and *in vitro* embryo production

This study comprised two experiments evaluating FF-sEVs supplementation during IVM. Experiment 1 examined group-cultured oocytes supplemented with 0 (control), 5, 10, 25, or 50 μg FF-sEVs protein/mL, while Experiment 2 assessed individually cultured oocytes supplemented with 0 (control), 6.5, 12.5, or 25 μg FF-sEVs protein/mL. To do so, FF-sEVs protein concentrations from each heifer were quantified using a NanoDrop ND-1000 spectrophotometer (Thermo Fisher Scientific, Wilmington, DE, USA) at 280 nm. Individual FF-sEVs stock solutions were prepared by diluting FF-sEVs in PBS containing 0.1% BSA (Sigma A8806). To ensure equal contributions from all heifers, stock solutions were pooled proportionally and stored as 0.5 mL aliquots at −80°C. Pooled FF-sEVs aliquots were thawed and serially diluted in pre-equilibrated maturation media (38.5°C, 5% CO₂) to achieve target concentrations.

Ovaries were collected from a local slaughterhouse, externally disinfected with 96% ethanol, and rinsed in warm (37°C) physiological saline containing kanamycin (50 μg/mL). Cumulus-oocyte complexes were aspirated from antral follicles (4–8 mm diameter) using an 18-gauge needle and 10 mL syringe. Cumulus oocyte complexes with uniformly granulated cytoplasm and ≥3 compact cumulus cell layers were selected for IVM, *in vitro* fertilization (IVF), and *in vitro* culture (IVC) under group and individual conditions, as previously described (26) with minor modifications.

#### Group culture system

For IVM, batches of 60 COCs were cultured in 500 μL TCM-199 supplemented with 20 ng/mL epidermal growth factor, 50 μg/mL gentamycin, and FF-sEVs at specified concentrations (0, 5, 10, 25, or 50 μg FF-sEVs protein/mL). After 22 h (38.5°C, 5% CO₂), matured oocytes were fertilized with frozen–thawed bull spermatozoa purified via Percoll gradient (45%/90%). Sperm concentration was adjusted to 1 × 10^6^/mL in IVF-TALP (bicarbonate-buffered Tyrode’s medium with 6 mg/mL BSA [Sigma A8806] and 20 μg/mL heparin). Fertilization occurred in 500 μL IVF-TALP for 21 h (38.5°C, 5% CO₂). Excess sperm and cumulus cells were removed by vortexing, and zygotes were transferred to 50 μL droplets of synthetic oviductal fluid (SOF) containing 0.4% BSA (Sigma A9647) and ITS (5 μg/mL insulin, 5 μg/mL transferrin, 5 ng/mL selenium). Groups of 25 zygotes were cultured under paraffin oil (SAGE, Cooper Surgical) at 38.5°C for 8 days (5% CO₂, 5% O₂, 90% N₂).

#### Individual culture system

Media compositions mirrored group culture conditions, but all steps (IVM, IVF, IVC) were performed in single-COC/zygote droplets (35, 36). For IVM, individual COCs were matured in 20 μL droplets of TCM-199 (supplemented with 0, 6.5, 12.5, or 25 μg FF-sEVs protein/mL) under paraffin oil (7.5 mL in 60 × 15 mm Petri dishes). Post-IVM, fertilization was conducted in 20 μL IVF-TALP droplets (1 × 10^6^ sperm/mL) for 21 h. Cumulus-free zygotes were transferred to 20 μL SOF droplets and cultured individually for 8 days under identical gas and temperature conditions as group culture.

Cleavage rate was calculated at 45 h post-insemination as the percentage of cleaved embryos relative to presumed zygotes. Blastocyst rates were determined on days 7 and 8 post-insemination as the percentage of blastocysts relative to presumed zygotes.

### Differential apoptotic staining of blastocysts

*In vitro*-produced day 8 blastocysts were fixated in 4% paraformaldehyde for 20 min at room temperature (RT) and stored at 4°C until further processing. Differential staining was performed using the protocol adapted from Wydooghe et al. (37). Blastocysts were permeabilized in 0.5% Triton X-100 and 0.05% Tween-20 in PBS for 1 h at RT. DNA denaturation was achieved by treating samples with 2 N HCl (Tritipur®, Merck, Germany; Cat. 1.09063.1000, Lot HC98019763) for 20 min at RT, followed by neutralization in 100 mM Tris–HCl (pH 8.5) for 10 min. Embryos were then incubated overnight at 4°C in blocking solution (10% goat serum, 0.5% BSA in PBS; Gibco®, UK; Cat. 0000333482).

For lineage-specific staining, blastocysts were incubated overnight at 4°C with a ready-to-use mouse anti-CDX2 monoclonal antibody (Biogenex, San Ramon, CA, USA) to label the inner cell mass (ICM). After washing, samples were incubated overnight at 4°C with rabbit anti-active caspase-3 antibody (0.768 μg/mL in blocking solution; Cell Signaling Technology, Leiden, Netherlands; Cat. 9,664) to detect apoptotic cells. Negative controls omitted primary antibodies. Secondary staining involved sequential incubation with goat anti-mouse Texas Red-conjugated antibody (20 μg/mL; Molecular Probes, Merelbeke, Belgium; Cat. T-862) and goat anti-rabbit FITC-conjugated antibody (10 μg/mL; Molecular Probes; Cat. F-2765), each for 1 h at RT. Blastocysts were counterstained with Hoechst 33342 (50 μg/mL in PBS/0.1% BSA; Molecular Probes; Cat. H3570) for 20 min at RT to label all nuclei and mounted in DABCO antifade medium (≤10 blastocysts per slide).

Mounted blastocysts were imaged using a confocal microscope (Leica Microsystems GmbH, Wetzlar, Germany). Trophectoderm (TE) cells were identified by Texas Red fluorescence, while ICM cells were demarcated by CDX2 labelling. Total cell number (TCN) was calculated as the sum of TE and ICM nuclei stained with Hoechst 33342. Apoptotic cells (AC) were quantified based on FITC-positive caspase-3 signal. Ratios of ICM/TCN and apoptotic cell ratio (ACR; AC/TCN) were derived from these counts. Image overlays were generated using ImageJ software v1.52d (National Institutes of Health, USA).

### Statistical analyses

All statistical analyses were conducted in RStudio (v4.2.1; R Core Team, Vienna, Austria) with the zygote/embryo as the experimental unit. Generalized linear mixed-effects models were fitted to evaluate the impact of FF-sEVs supplementation at varying concentrations (group culture: 0, 5, 10, 25, and 50 μg protein/mL; individual culture: 0, 6.5, 12.5, and 25 μg protein/mL) on embryo development outcomes, including cleavage rate and blastocyst formation (days 7 and 8 post-insemination). Similarly, mixed linear regression models were used to assess the effects of FF-sEVs supplementation on blastocyst differential staining parameters (TE, ICM, TCN, ICM/TCN ratio, AC, and ACR). Replicate was included as a random effect in all models. Assumptions of homoscedasticity, linearity, and normality were verified via scatterplots of studentized residuals, linear predictor diagnostics, and Shapiro–Wilk tests, respectively. Continuous variables violating normality assumptions (*p* < 0.05) were transformed using square root-, log2-, or log10-transformations, after which residuals met normality criteria (Shapiro–Wilk *p* > 0.05). Pairwise comparisons between FF-sEVs treatment groups were performed using Tukey’s *post hoc* test, with results reported as least squares means ± standard errors. Analyses utilized the R packages lme4, multcomp, and multcompView, and statistical significance was defined as *p* ≤ 0.05.

## Results

**Figure 1:**
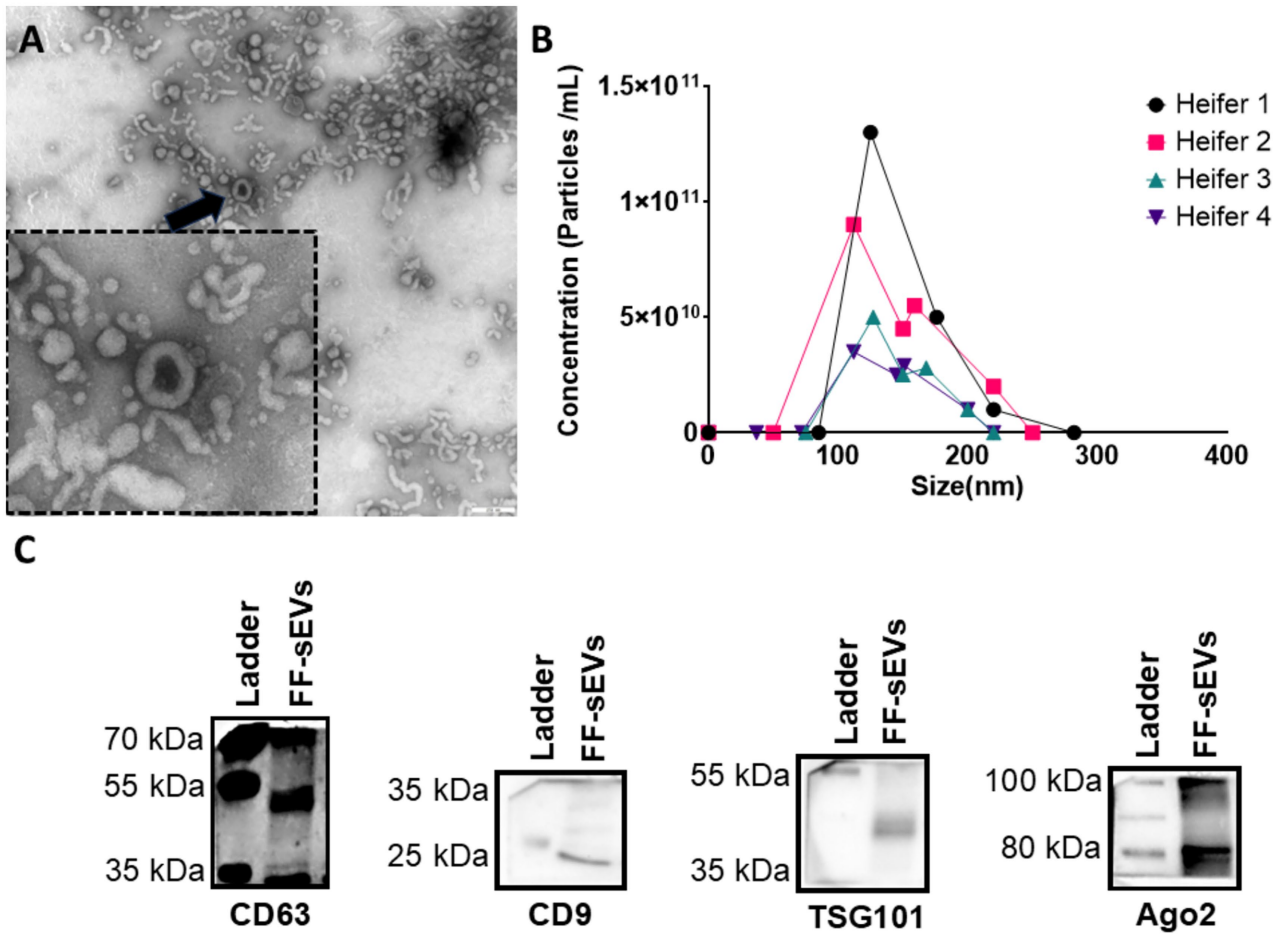
Characterization of follicular fluid-derived small extracellular vesicles (FF-sEVs) from nulliparous heifers isolated by OptiPrep™ density gradient ultracentrifugation. **(A)** Transmission electron microscopy image of FF-sEVs, showing vesicles with diameters ranging from 80 to 120 nm. The black arrow indicates an intact, spherical FF-sEV with a visible bilayer membrane. **(B)** Nanoparticle tracking analysis line plots displaying the particle concentration and size distribution of FF-sEVs from each heifer, demonstrating similar size profiles among samples. The mean particle size of 165 nm is consistent with the characteristics of small EVs. **(C)** Western blot analysis of FF-sEVs, showing positive expression of EV-specific markers CD63 (42 kDa), CD9 (25 kDa), and TSG101 (49 kDa), and negative expression of AGO2 (87 kDa), confirming EV enrichment.

**Figure 2:**
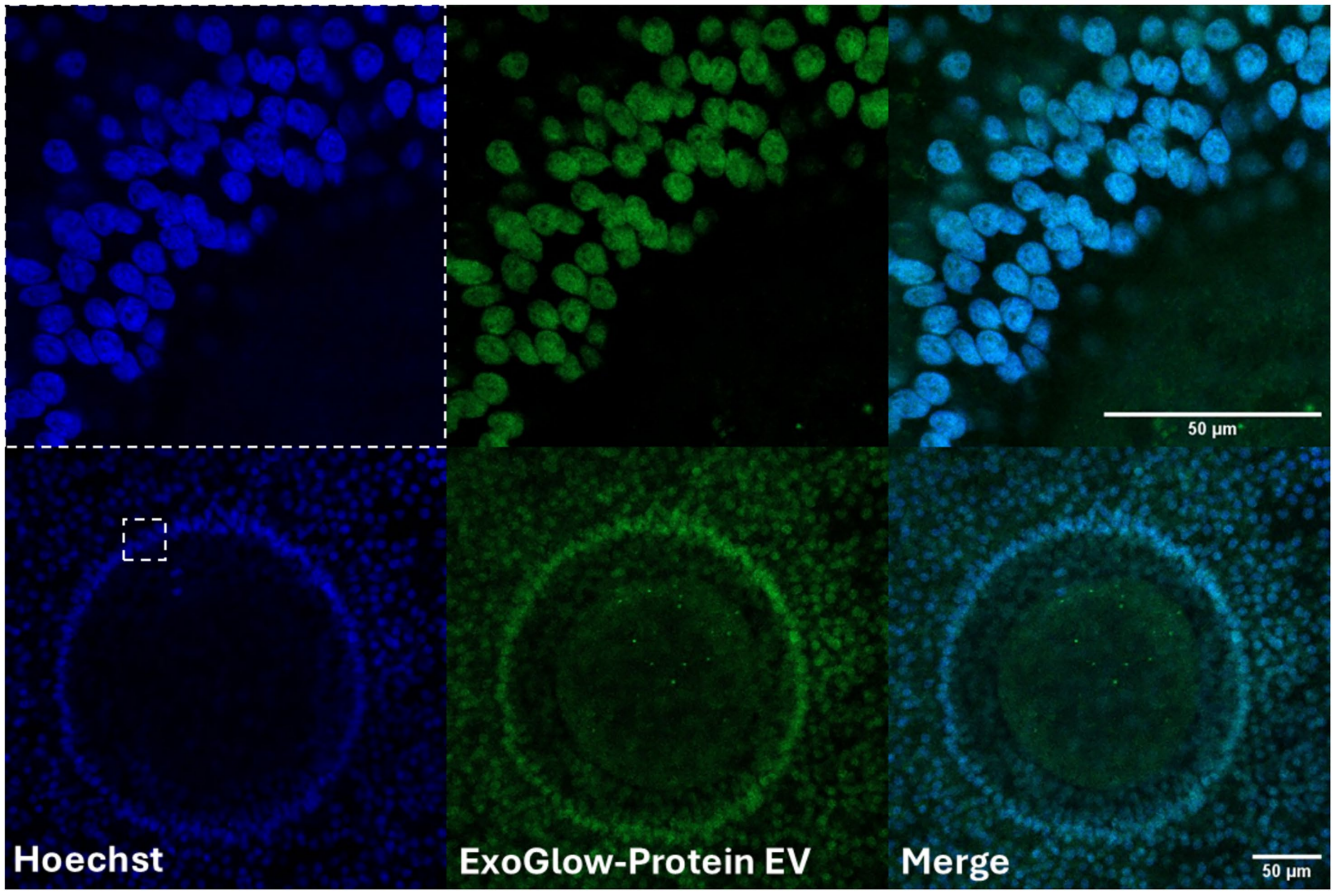
Confocal microscopy images revealed the presence of labeled follicular fluid-derived small extracellular vesicles (FF-sEVs) within both the ooplasm of the oocyte and the cytoplasm of surrounding granulosa cells. The uptake of fluorescently FF-sEVs by cumulus-oocytes compexes (COCs) was evaluated following 22 h of co-incubation in maturation medium. Follicular fluid-sEVs were labeled using the ExoGlow-Protein EV Labeling Kit and COCs were counterstained using Hoechst 33342.

Characterization of FF-sEVs revealed bilayer, spherical particles consistent with EV morphology, as observed by TEM (Figure 1A). Nanoparticle tracking analysis indicated mean ± SD particle sizes of 160.6 ± 55.1 nm (heifer 1), 164.6 ± 73.8 nm (heifer 2), 170.1 ± 80.4 nm (heifer 3), and 157.0 ± 61.4 nm (heifer 4) (Figure 1B). The protein concentrations of these samples measured 30.34, 28.13, 23.81, and 20.93 μg/mL, respectively. Western blot, performed on a FF-sEVs pool from the four heifers, confirmed the presence of EV-specific markers (CD9, CD63, and TSG101) (Figure 1C). The uptake of fluorescently labelled FF-sEVs by COCs was evaluated following 22 h of co-incubation in maturation medium. Confocal microscopy images acquired after the maturation period revealed the presence of labelled FF-sEVs within both the ooplasm of the oocyte and the cytoplasm of surrounding cumulus cells (Figure 2).

Embryo development parameters for oocytes/zygotes cultured in groups (*n* = 2,062 COCs in 8 replicates) are summarized in Table 1. Cleavage rate and day 7 blastocyst formation did not differ among groups (*p* > 0.38). However, the day 8 blastocyst rate in the control group was higher compared to COCs supplemented with FF-sEVs at 10, 25, or 50 μg/mL (*p* < 0.02), while no difference was observed between the control and the 5 μg/mL FF-sEVs group (*p* > 0.79).

**Table 1:** Embryo development rate in group maturation in the presence of follicular fluid-derived small extracellular vesicles (FF-sEVs).

Group	No. of oocytes	Cleavage	Day 7 blastocyst	Day 8 blastocyst
Control	410	80.3 ± 2.6	18.0 ± 2.0	35.8 ± 2.6^a^
sEVs 5 μg/mL	400	77.1 ± 2.9	18.7 ± 2.0	32.0 ± 2.5^ab^
sEVs 10 μg/mL	417	77.2 ± 2.8	17.0 ± 1.9	26.0 ± 2.3^bc^
sEVs 25 μg/mL	416	75.0 ± 3.0	17.5 ± 1.9	25.2 ± 2.3^bc^
sEVs 50 μg/mL	419	75.6 ± 2.9	14.8 ± 1.8	22.9 ± 2.2^c^

Cleavage, day 7, and day 8 blastocyst rates expressed as a percentage of presumed zygotes. Within 8 replicates, maturation media of group cultured oocytes (*n* = 60 oocytes) were supplemented with FF-sEVs to 0 (control), 5, 10, 25, and 50 μg/mL sEV-protein. Results are expressed as least square means ± standard errors. Within a column, different superscripts ^a,b,c^ indicate significant differences (*p* < 0.05).

Embryo development parameters (*n* = 540 COCs in 8 replicates) of individually cultured oocytes/zygotes are presented in Table 2. Cleavage rates and day 7 blastocyst production did not differ (*p* > 0.19) between FF-sEVs-supplemented groups (6.5, 12.5, or 25 μg/mL) and the control. However, supplementation with 6.5 μg/mL FF-sEVs in individual cultures improved day 8 blastocyst rates compared to all other groups (*p* < 0.02). No differences in day 8 blastocyst production were observed between the 12.5 μg/mL, 25 μg/mL, and control groups (*p* > 0.95).

**Table 2:** Embryo development rate in individual maturation in the presence of follicular fluid-derived small extracellular vesicles (FF-sEVs).

Group	No. of oocytes	Cleavage	Day 7 blastocyst	Day 8 blastocyst
Control	136	80.1 ± 3.4	14.7 ± 3.0	23.5 ± 3.6^a^
sEVs 6.5 μg/mL	136	88.2 ± 2.7	24.3 ± 3.6	42.6 ± 4.2^b^
sEVs 12.5 μg/mL	135	77.0 ± 3.6	14.8 ± 3.0	22.2 ± 3.5^a^
sEVs 25 μg/mL	133	77.4 ± 3.6	16.5 ± 3.2	26.3 ± 3.8^a^

Cleavage, day 7, and day 8 blastocyst rates expressed as a percentage of presumed zygotes. Within 8 replicates, maturation media of individually cultured oocytes were supplemented with FF-sEVs to 6.5, 12.5, and 25 μg/mL sEV-protein. Results are expressed as least square means ± standard errors. Within a column, different superscripts ^a,b^ indicate significant differences (*p* < 0.05).

Differential apoptotic staining of day 8 blastocysts (Table 3) demonstrated that supplementation of group-cultured oocytes/zygotes with 25 or 50 μg/mL FF-sEVs yielded blastocysts with lower TCN compared to those supplemented with 5 or 10 μg/mL FF-sEVs or the control group (*p* < 0.001). Notably, COCs supplemented with 50 μg/mL FF-sEVs produced blastocysts with reduced ICM counts and lower ICM/TCN ratios compared to all other groups (*p* < 0.001). Supplementation with 5 μg/mL FF-sEVs yielded blastocysts with the highest ICM/TCN ratio compared to all other groups (*p* < 0.01). No differences were observed in the ACR across experimental groups (*p* > 0.14).

**Table 3:** Effect of supplementation of different concentrations of follicular fluid-derived small extracellular vesicles (FF-sEVs) during *in vitro* group oocyte maturation on embryo quality.

Group	No. of blastocysts	Cell numbers				Ratios (%)	
		TCN	ICM	TE	AC	ICM/TCN	AC/TCN
Control	143	143 ± 1.7^a^	72.8 ± 1.2^a^	70.5 ± 1.1^ab^	4.0 ± 0.12^a^	50.5 ± 5.4^a^	2.7 ± 0.06
sEVs 5 μg/mL	128	140 ± 1.8^a^	74.8 ± 1.2^a^	65.2 ± 1.2^c^	4.1 ± 0.13^a^	53.5 ± 5.7^b^	2.8 ± 0.07
sEVs 10 μg/mL	109	140 ± 2.0^a^	65.2 ± 1.4^c^	74.4 ± 1.2^b^	4.1 ± 0.14^a^	46.4 ± 6.2^c^	2.8 ± 0.07
sEVs 25 μg/mL	105	123 ± 2.0^b^	55.3 ± 1.4^d^	67.9 ± 1.3^bc^	3.7 ± 0.15^ab^	44.2 ± 6.3^c^	2.9 ± 0.08
sEVs 50 μg/mL	96	119 ± 1.2^b^	44.2 ± 1.4^e^	74.4 ± 1.3^b^	3.3 ± 1.15^b^	36.8 ± 6.6^d^	2.7 ± 0.08

Total cell numbers (TCN), trophectoderm cells (TE), inner cell mass (ICM), apoptotic cells (AC), and their respective ratios of day 8 blastocysts subjected to a differential/apoptotic fluorescent staining. Within 8 replicates, maturation media of group cultured oocytes (*n* = 60) were supplemented with FF-sEVs to 0 (control), 5, 10, 25, and 50 μg/mL sEV-protein. Results are expressed as least square means ± standard errors. Within a column, different superscripts ^a,b,c,d^ indicate significant differences (*p* < 0.05).

**Figure 3:**
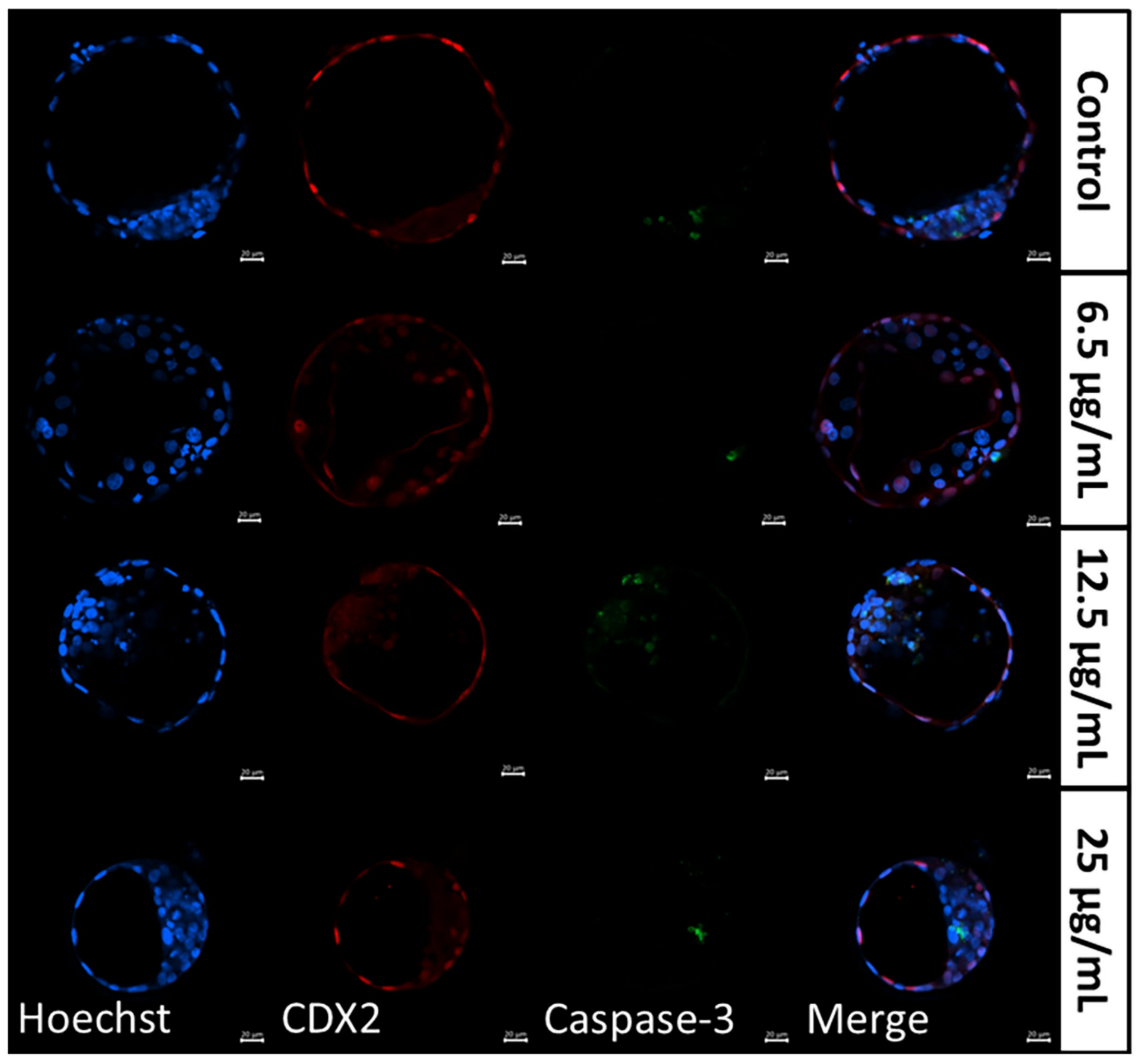
Representative images of differentially stained day 8 blastocysts resulting from individually matured oocytes, supplemented with follicular fluid-derived small extracellular vesicles (FF-sEVs) to 6.5, 12.5, and 25 μg/mL EV-protein. Hoechst 33342 aimed to stain nuclei in blue, CDX2 stained the trophectoderm cells in red, and Caspase-3 labelled apoptotic cells in green.

Differential apoptotic staining of day 8 blastocysts (Table 4 and Figure 3) revealed that individual culture with 6.5 μg/mL FF-sEVs produced blastocysts with higher TCN and ICM counts compared to all other groups (*p* < 0.001). The ICM/TCN ratio was also greater in the 6.5 μg/mL FF-sEVs group (*p* < 0.001). In contrast, TE cell counts and AC numbers showed no differences across groups (*p* > 0.34). Notably, the ACR was lower in the 6.5 μg/mL FF-sEVs group than in all other groups (*p* < 0.001).

**Table 4:** Effect of supplementation of different concentrations of follicular fluid-derived extracellular vesicles (FF-sEVs) during *in vitro* individual oocyte maturation on embryo quality.

Group	No. of blastocysts	Cell numbers				Ratios (%)	
		TCN	ICM	TE	AC	ICM/TCN	AC/TCN
Control	31	100.6 ± 1.2^a^	27.3 ± 0.8^a^	73.2 ± 1.7	3.9 ± 0.16	27.2 ± 1.0^a^	3.9 ± 0.13^a^
sEVs 6.5 μg/mL	51	125.3 ± 1.1^b^	51.9 ± 0.7^b^	73.3 ± 1.7	3.7 ± 0.15	41.6 ± 0.9^b^	2.9 ± 0.12^b^
sEVs 12.5 μg/mL	25	99.6 ± 1.2^a^	26.0 ± 0.8^a^	73.5 ± 1.7	3.8 ± 0.17	26.2 ± 1.0^a^	3.8 ± 0.14^a^
sEVs 25 μg/mL	27	99.8 ± 1.2^a^	27.4 ± 0.8^a^	72.4 ± 1.7	3.8 ± 0.17	27.4 ± 1.0^a^	3.8 ± 0.13^a^

Total cell numbers (TCN), trophectoderm cells (TE), inner cell mass (ICM), apoptotic cells (AC), and their respective ratios of day 8 blastocysts subjected to a differential/apoptotic fluorescent staining. Within 8 replicates, maturation media of individually cultured oocytes were supplemented with FF-sEVs to 6.5, 12.5, and 25 μg/mL sEV-protein. Results are expressed as least square means ± standard errors. Within a column, different superscripts ^a,b^ indicate significant differences (*p* < 0.05).

## Discussion

We investigated the role of FF-sEVs in optimizing *in vitro* embryo development, specifically examining their dose-dependent effects in group and individual culture systems. We successfully isolated FF-sEVs from dominant follicles of the first follicular wave and demonstrated their internalization by oocytes and granulosa cells during IVM. Intriguingly, while group-cultured COCs and zygotes showed no developmental improvement with FF-sEVs supplementation, adverse outcomes emerged at higher concentrations (blastocyst yield and TCN were reduced at FF-sEVs doses ≥25 μg/mL). This inhibitory effect may reflect competitive interference between exogenous FF-sEVs and endogenous embryokines actively released during group culture within the maturation medium. Individual COC cultures supplemented with a low FF-sEVs concentration (6.5 μg/mL) exhibited enhanced developmental competence, achieving day 8 blastocyst production rates comparable to group-cultured counterparts. However, increasing FF-sEVs doses in individual systems yielded no further benefit, underscoring the existence of a narrow therapeutic window. These results suggest that calibrated FF-sEVs supplementation can compensate for the absence of paracrine signaling in individual COC cultures. By replicating cell-to-cell communication pathways, FF-sEVs offer a novel strategy to refine ART, particularly in single-embryo culture formats where physiological interactions are limited.

Characterization of FF-sEVs via NTA revealed a mean size of 160–170 nm, aligning with the typical size range of exosomes and microvesicles as reported in bovine FF (16, 38) and in the FF other domestic species (19, 20, 39). While exosomes originate from endocytic pathways and are constitutively secreted, microvesicles form via plasma membrane budding during cellular activation (40). Current nomenclature classifies vesicles within this size range as “small EVs” (31) and, regardless of biogenesis, we focused on their embryotropic effects. Protein concentrations measured using Nanodrop (20–30 μg/mL) were also consistent with prior studies (16, 41). Asaadi et al. (16) found that FF-sEVs isolated via ODG UC exhibited lower protein concentrations than those isolated by size-exclusion chromatography (SEC), as ODG UC minimizes contaminating proteins (e.g., lipoproteins). However, ultracentrifugation may partially compromise sEVs integrity due to high gravitational forces, leading to sEVs loss (32, 33). Despite this limitation, ODG UC better preserves sEVs’ functional cargo (e.g., proteins, lipids, and RNAs) compared to alternative methods (e.g., multistep ultracentrifugation or SEC) (28, 33). Supporting this, our previous work (16) demonstrated that ODG UC-isolated FF-sEVs from preovulatory follicles enhanced embryo production, whereas SEC-isolated FF-sEVs showed no effect. These findings confirm the superior integrity and functionality of ODG UC-isolated sEVs, likely attributable to reduced contamination and preserved bioactive cargo.

The biological activity of sEVs depends on their internalization by recipient cells. In domestic species such as cattle, horses, and cats, FF-sEVs have been shown to interact with COCs, with uptake documented in cumulus cells during IVM (20, 23, 41). More recently, in an equine IVM model it was also demonstrated that supplementing FF-sEVs to maturation media for 38 h resulted in sEVs absorption by granulosa cells as well as oocytes per se (34). Similarly, in the present study, internalization of FF-sEVs was confirmed by their localization within the ooplasm, delineated by the distinct circular boundary of the oolemma, and within granulosa cells, as evidenced by labelled FF-sEVs clustered in the cytoplasm. While these findings highlight the capacity of COCs to internalize FF-sEVs, the precise physiological mechanisms governing this process remain unclear. In this regard, emerging evidence suggests that FF-sEVs may enhance oocyte maturation through dual pathways: (1) modulating granulosa cell function by delivering bioactive molecules (e.g., miRNAs, proteins) that regulate steroidogenesis, apoptosis, and cell-cycle progression (42, 43) and (2) directly influencing oocyte competence by transferring metabolic regulators (e.g., ATP-generating enzymes) and anti-apoptotic factors (44). These mechanisms collectively suggest that FF-sEVs act as critical mediators of cell–cell communication, bridging cell support and direct oocyte programming to optimize maturation outcomes.

Building on the findings of Asaadi et al. (16), who demonstrated improved embryo development with 12.5 μg/mL FF-sEVs supplementation during group oocyte maturation, the present study tested concentrations around that benchmark (0, 5, 10, 25, and 50 μg protein/mL) to assess responses below, near, and above this reference, while acknowledging that dose–response relationships may not be linear. In contrast, for the individual culture system, no prior study had supplemented FF-sEVs during maturation. However, our previous work showed that supplementing FF at 5 and 10% (v/v) in the maturation medium of group-cultured COCs improved several blastocyst quality parameters compared with controls (35). While in individually cultured COCs, only 5% FF improved blastocyst quality parameters as well as blastocyst production (35). Assuming that lower FF volumes contain proportionally fewer FF-sEVs, concentrations for the individual culture system were chosen within and below Asaadi et al.’s (16) therapeutic window (6.5, 12.5, and 25 μg/mL). This approach was also considered logical because IVM in the group culture system involves 60 COCs per well, making higher supplementation concentrations more appropriate than in the individual culture setting, where a single COC is present. Interestingly, because improvements in IVP systems often arise from mimicking *in vivo* conditions, we conducted a preliminary study (data not shown) in which we tested higher FF-sEVs concentrations in the maturation medium (12.5, 125, and 1,250 μg protein/mL) to more closely approximate those present within follicles. However, concentrations ≥125 μg protein/mL led to a marked reduction in blastocyst production (<10%).

The observed discrepancies between our findings and earlier studies, such as the developmental benefits of 12.5 μg/mL FF-sEVs in group cultures reported by Asaadi et al. (16), may arise from differences in the follicular origin of sEVs (16). Specifically, FF-sEVs in the present study were isolated from presumably growing follicles during the luteal phase of the oestrous cycle (dominant follicles of ~8 mm), whereas Asaadi et al. (16) utilized sEVs derived from pre-ovulatory follicles (~15 mm). In a previous study, da Silveira et al. (23) demonstrated that small-follicle sEVs enhance blastocyst rates by modulating metabolic and epigenetic pathways, including DNMT3A expression, whereas pre-ovulatory follicle sEVs exhibit neutral or inhibitory effects. These functional differences likely reflect the divergent follicular microenvironments: developing follicles are rich in growth factors such as vascular endothelial growth factor (VEGF), insulin-like growth factor 1 (IGF-1), and fibroblast growth factor (FGF), which are known to support oocyte maturation and early embryonic development (45, 46). In contrast, pre-ovulatory follicles are primed for ovulation, a process mediated by proinflammatory factors like tumour necrosis factor-alpha (TNF-*α*), interleukin-6 (IL-6), and prostaglandins (47, 48). While these mediators are not inherently detrimental to oocytes, they are physiologically restricted to the final stages of maturation (e.g., meiotic resumption), where they orchestrate follicle rupture and cumulus expansion. We propose that group-cultured oocytes and zygotes inherently produce sufficient endogenous growth factors to sustain development. Consequently, supplementing this self-sufficient microenvironment with FF-sEVs derived from developing follicles (already rich in developmental cues) may disrupt equilibrium, leading to suboptimal IVM conditions. This hypothesis aligns with our observation that higher FF-sEVs concentrations (e.g., ≥25 μg/mL) impaired blastocyst development and quality in group culture conditions. Conversely, low doses of pre-ovulatory FF-sEVs (e.g., 12.5 μg/mL) may act synergistically during late-stage oocyte maturation, providing targeted support for processes like cytoplasmic maturation or cumulus matrix remodeling. Collectively, these findings underscore the functional significance of follicular stage in FF-sEVs activity.

Supplementation with 6.5 μg/mL FF-sEVs significantly enhanced blastocyst development and quality in individually cultured COCs compared to both higher FF-sEVs concentrations (12.5 and 25 μg/mL) and non-supplemented controls. Interestingly, COCs treated with 6.5 μg/mL FF-sEVs achieved blastocyst rates comparable to group culture systems, though their blastocysts exhibited lower TCN (125 vs. 143 cells, respectively). This disparity implies that while FF-sEVs effectively compensate for deficiencies in IVM by delivering critical developmental mediators (e.g., miRNA, proteins, and lipids), post-fertilization culture conditions may limit blastocyst quality. The 6.5 μg/mL dose appears to replicate the group culture environment by providing a balanced cocktail of embryokines-like factors (likely present in the FF-sEVs cargo) that mimics the autocrine/paracrine signalling of group-cultured COCs. However, the present study’s key limitation lies in its inability to isolate the contributions of specific sEVs components (e.g., miRNAs vs. proteins) to the observed improvements. Future studies should employ cargo-depletion approaches (e.g., RNase-treated EVs, lipid extraction) or single-omics analyses to identify the active agents. Supplementing individual culture systems with oviductal or endometrial sEVs during post-fertilization phases could help elucidate their role in enhancing blastocyst quality, particularly by mimicking physiological embryo-maternal communication. A further limitation is that the number of experimental replicates was not determined using an *a priori* sample size calculation. Nevertheless, the increase in blastocyst production in the individual culture system with 6.5 μg/mL FF-sEVs supplementation during maturation was substantial.

## Conclusion

Supplementation with 6.5 μg/mL FF-sEVs in individually cultured COCs enhanced day 8 blastocyst rates, TCN, and ICM, while also reducing AR, compared to both higher FF-sEVs concentrations (12.5 and 25 μg/mL) and non-supplemented controls. In contrast, group-cultured COCs and zygotes showed no developmental improvements with FF-sEVs supplementation, and adverse outcomes emerged at higher concentrations: blastocyst yield and TCN were reduced at FF-sEVs doses ≥10 μg/mL. Calibrated FF-sEVs supplementation appears to compensate for the lack of paracrine signaling in individual cultures. The negative effects in group-cultured COCs may result from competitive interference between exogenous FF-sEVs and endogenous embryokines in the maturation medium. The identification of potentially conserved RNAs, proteins, or lipids in FF-sEVs across mammalian species, including humans, could support the development of fully synthetic media to enhance and optimize IVP. In the present study, however, the specific FF-sEVs components responsible for the observed improvements in blastocyst production remain unknown.

## Data Availability

The raw data supporting the conclusions of this article will be made available by the authors, without undue reservation.

## References

[1] Fazeli A Godakumara K. The evolving roles of extracellular vesicles in embryo-maternal communication. Commun Biol. (2024) 7:754. doi: 10.1038/s42003-024-06442-9, PMID: 38906986 PMC11192758

[2] Aleksejeva E Zarovni N Dissanayake K Godakumara K Vigano P Fazeli A. Extracellular vesicle research in reproductive science: paving the way for clinical achievements. Biol Reprod. (2022) 106:408–24. doi: 10.1093/biolre/ioab245, PMID: 34982163

[3] Xue Y Zheng H Xiong Y Li K. Extracellular vesicles affecting embryo development in vitro: a potential culture medium supplement. Front Pharmacol. (2024) 15:1366992. doi: 10.3389/fphar.2024.1366992, PMID: 39359245 PMC11445000

[4] Fan W Qi Y Wang Y Yan H Li X Zhang Y. Messenger roles of extracellular vesicles during fertilization of gametes, development and implantation: recent advances. Front Cell Dev Biol. (2023) 10:1079387. doi: 10.3389/fcell.2022.1079387, PMID: 36684431 PMC9849778

[5] Machtinger R Baccarelli AA Wu H. Extracellular vesicles and female reproduction. J Assist Reprod Genet. (2021) 38:549–57. doi: 10.1007/s10815-020-02048-2, PMID: 33471231 PMC7910356

[6] Gurunathan S Kang MH Song H Kim NH Kim JH. The role of extracellular vesicles in animal reproduction and diseases. J Anim Sci Biotechnol. (2022) 13:62. doi: 10.1186/s40104-022-00715-1, PMID: 35681164 PMC9185900

[7] Chen K Liang J Qin T Zhang Y Chen X Wang Z. The role of extracellular vesicles in embryo implantation. Front Endocrinol (Lausanne). (2022) 13:809596. doi: 10.3389/fendo.2022.809596, PMID: 35154016 PMC8831238

[8] de Alcântara-Neto AS Cuello C Uzbekov R Bauersachs S Mermillod P Almiñana C. Oviductal extracellular vesicles enhance porcine in vitro embryo development by modulating the embryonic transcriptome. Biomolecules. (2022) 12:1300. doi: 10.3390/biom12091300, PMID: 36139139 PMC9496104

[9] Yáñez-Mó M Siljander PR-M Andreu Z Bedina Zavec A Borràs FE Buzas EI. Biological properties of extracellular vesicles and their physiological functions. J Extracell Vesicles. (2015) 4:27066. doi: 10.3402/jev.v4.27066 25979354 PMC4433489

[10] Kalluri R LeBleu VS. The biology, function, and biomedical applications of exosomes. Science. (2020) 367:eaau6977. doi: 10.1126/science.aau6977, PMID: 32029601 PMC7717626

[11] Gregory CD Rimmer MP. Extracellular vesicles arising from apoptosis: forms, functions, and applications. J Pathol. (2023) 260:592–608. doi: 10.1002/path.6138, PMID: 37294158 PMC10952477

[12] Tetta C Ghigo E Silengo L Deregibus MC Camussi G. Extracellular vesicles as an emerging mechanism of cell-to-cell communication. Endocrine. (2013) 44:11–9. doi: 10.1007/s12020-012-9839-0, PMID: 23203002 PMC3726927

[13] Tesfaye D Hailay T Salilew-Wondim D Hoelker M Bitseha S Gebremedhn S. Extracellular vesicle mediated molecular signaling in ovarian follicle: implication for oocyte developmental competence. Theriogenology. (2020) 150:70–4. doi: 10.1016/j.theriogenology.2020.01.075, PMID: 32088041

[14] Zeng Y Qiu Y Jiang W Shen J Yao X He X. Biological features of extracellular vesicles and challenges. Front Cell Dev Biol. (2022) 10:816698. doi: 10.3389/fcell.2022.816698, PMID: 35813192 PMC9263222

[15] da Silveira JC Carnevale EM Winger QA Bouma GJ. Regulation of ACVR1 and ID2 by cell-secreted exosomes during follicle maturation in the mare. Reprod Biol Endocrinol. (2014) 12:44. doi: 10.1186/1477-7827-12-44, PMID: 24884710 PMC4045866

[16] Asaadi A Dolatabad NA Atashi H Raes A Van Damme P Hoelker M. Extracellular vesicles from follicular and Ampullary fluid isolated by density gradient ultracentrifugation improve bovine embryo development and quality. Int J Mol Sci. (2021) 22:578. doi: 10.3390/ijms22020578, PMID: 33430094 PMC7826877

[17] Gebremedhn S Gad A Ishak GM Menjivar NG Gastal MO Feugang JM. Dynamics of extracellular vesicle-coupled microRNAs in equine follicular fluid associated with follicle selection and ovulation. Mol Hum Reprod. (2023) 29:gaad009. doi: 10.1093/molehr/gaad009, PMID: 36852862 PMC10321592

[18] Machtinger R Rodosthenous R Mansour A Adir M Racowsky C Hauser R. Mirnas isolated from extracellular vesicles in follicular fluid and oocyte development potential. Fertil Steril. (2015) 104:e54. doi: 10.1016/j.fertnstert.2015.07.162

[19] Gabryś J Gurgul A Szmatoła T Kij-Mitka B Andronowska A Karnas E. Follicular fluid-derived extracellular vesicles influence on in vitro maturation of equine oocyte: impact on cumulus cell viability, expansion and transcriptome. Int J Mol Sci. (2024) 25:3262. doi: 10.3390/ijms25136812 38542236 PMC10970002

[20] de Almeida Monteiro Melo Ferraz M Fujihara M Nagashima JB Noonan MJ Inoue-Murayama M Songsasen N. Follicular extracellular vesicles enhance meiotic resumption of domestic cat vitrified oocytes. Sci Rep. (2020) 10:8619. doi: 10.1038/s41598-020-65497-w, PMID: 32451384 PMC7248092

[21] Hung WT Navakanitworakul R Khan T Zhang P Davis JS McGinnis LK. Stage-specific follicular extracellular vesicle uptake and regulation of bovine granulosa cell proliferation. Biol Reprod. (2017) 97:644–55. doi: 10.1093/biolre/iox106, PMID: 29025042 PMC6248580

[22] Hung WT Hong X Christenson LK McGinnis LK. Extracellular vesicles from bovine follicular fluid support cumulus expansion. Biol Reprod. (2015) 93:117. doi: 10.1095/biolreprod.115.132977, PMID: 26423123 PMC4712005

[23] da Silveira JC Andrade GM del Collado M Sampaio RV Sangalli JR Silva LA. Supplementation with small-extracellular vesicles from ovarian follicular fluid during in vitro production modulates bovine embryo development. PLoS One. (2017) 12:e0179451. doi: 10.1371/journal.pone.0179451, PMID: 28617821 PMC5472319

[24] Makieva S Saenz-de-Juano MD Almiñana C Bauersachs S Bernal-Ulloa S Xie M. Treatment of human oocytes with extracellular vesicles from follicular fluid during rescue in vitro maturation enhances maturation rates and modulates oocyte proteome and ultrastructure (2025). doi: 10.1101/2025.02.05.636623, PMC13037814 41924634

[25] Rizos D Ward F Duffy P Boland MP Lonergan P. Consequences of bovine oocyte maturation, fertilization or early embryo development in vitro versus in vivo: implications for blastocyst yield and blastocyst quality. Mol Reprod Dev. (2002) 61:234–48. doi: 10.1002/mrd.1153, PMID: 11803560

[26] Wydooghe E Heras S Dewulf J Piepers S Van den Abbeel E De Sutter P. Replacing serum in culture medium with albumin and insulin, transferrin and selenium is the key to successful bovine embryo development in individual culture. Reprod Fertil Dev. (2014) 26:717–24. doi: 10.1071/RD13043, PMID: 23711172

[27] Bunel A Jorssen EP Merckx E Leroy JL Bols PE Sirard MA. Individual bovine *in vitro* embryo production and cumulus cell transcriptomic analysis to distinguish cumulus-oocyte complexes with high or low developmental potential. Theriogenology. (2015) 83:228–37. doi: 10.1016/j.theriogenology 25442391

[28] Pavani KC Hendrix A Van Den Broeck W Couck L Szymanska K Lin X. Isolation and characterization of functionally active extracellular vesicles from culture medium conditioned by bovine embryos in vitro. Int J Mol Sci. (2018) 20:38. doi: 10.3390/ijms20010038, PMID: 30577682 PMC6337605

[29] Doherty EMO Wade MG Hill JL Boland MP. Effects of culturing bovine oocytes either singly or in groups on development to blastocysts. Theriogenology. (1997) 48:161–9. doi: 10.1016/S0093-691X(97)00199-4, PMID: 16728116

[30] Nishio M Hoshino Y Tanemura K Sato E. Effect of single-oocyte culture system on in vitro maturation and developmental competence in mice. Reprod Med Biol. (2014) 13:153–9. doi: 10.1007/s12522-014-0177-1, PMID: 29662372 PMC5892990

[31] Welsh JA Goberdhan DCI O’Driscoll L Buzas EI Blenkiron C Bussolati B. Minimal information for studies of extracellular vesicles (MISEV2023): from basic to advanced approaches. J Extracell Vesicles. (2024) 13:e12451. doi: 10.1002/jev2.12451 38326288 PMC10850029

[32] Van Deun J Mestdagh P Sormunen R Cocquyt V Vermaelen K Vandesompele J. The impact of disparate isolation methods for extracellular vesicles on downstream RNA profiling. J Extracell Vesicles. (2014) 3. doi: 10.3402/jev.v3.24858, PMID: 25317274 PMC4169610

[33] Pavani KC Lin X Hamacher J Van Den Broeck W Couck L Peelman L. The separation and characterization of extracellular vesicles from medium conditioned by bovine embryos. Int J Mol Sci. (2020) 21:2942. doi: 10.3390/ijms21082942, PMID: 32331414 PMC7215575

[34] Gabryś J Kij-Mitka B Sawicki S Kochan J Nowak A Łojko J. Extracellular vesicles from follicular fluid may improve the nuclear maturation rate of in vitro matured mare oocytes. Theriogenology. (2022) 188:116–24. doi: 10.1016/j.theriogenology.2022.05.022, PMID: 35689941

[35] Azari-Dolatabad N Raes A Pavani KC Asaadi A Angel-Velez D Van Damme P. Follicular fluid during individual oocyte maturation enhances cumulus expansion and improves embryo development and quality in a dose-specific manner. Theriogenology. (2021) 166:38–45. doi: 10.1016/j.theriogenology.2021.02.016, PMID: 33684781

[36] Azari-Dolatabad N Benedetti C Velez DA Montoro AF Sadeghi H Residiwati G. Oocyte developmental capacity is influenced by intrinsic ovarian factors in a bovine model for individual embryo production. Anim Reprod Sci. (2023) 249:107185. doi: 10.1016/j.anireprosci.2022.107185, PMID: 36610102

[37] Wydooghe E Vandaele L Beek J Favoreel H Heindryckx B De Sutter P. Differential apoptotic staining of mammalian blastocysts based on double immunofluorescent CDX2 and active caspase-3 staining. Anal Biochem. (2011) 416:228–30. doi: 10.1016/j.ab.2011.05.033, PMID: 21684250

[38] Lipinska P Smits K Van Soom A Pavani KC Warzych E. Follicular-fluid extracellular vesicles support energy metabolism of bovine oocytes, improving blastocyst development and quality. Biol Reprod. (2025) 113:109–26. doi: 10.1093/biolre/ioaf096, PMID: 40272384 PMC12260498

[39] Kang H Bang S Kim H Han A Miura S Park HS. Effects of follicular fluid-derived extracellular vesicles improve *in vitro* maturation and embryonic development of porcine oocytes. Korean J Vet Res. (2023) 63:e40. doi: 10.14405/kjvr.20230044

[40] Tannetta D Dragovic R Alyahyaei Z Southcombe J. Extracellular vesicles and reproduction–promotion of successful pregnancy. Cell Mol Immunol. (2014) 11:548–63. doi: 10.1038/cmi.2014.42, PMID: 24954226 PMC4220835

[41] da Silveira JC Veeramachaneni DNR Winger QA Carnevale EM Bouma GJ. Cell-secreted vesicles in equine ovarian follicular fluid contain miRNAs and proteins: a possible new form of cell communication within the ovarian Follicle1. Biol Reprod. (2012) 86:71. doi: 10.1095/biolreprod.111.093252, PMID: 22116803

[42] Six R Benedetti C Fan Y Guan X Gansemans Y Hedia M. Expression profile and gap-junctional transfer of microRNAs in the bovine cumulus-oocyte complex. Front Cell Dev Biol. (2024) 12:1404675. doi: 10.3389/fcell.2024.1404675, PMID: 39055654 PMC11269113

[43] Machtinger R Laurent LC Baccarelli AA. Extracellular vesicles: roles in gamete maturation, fertilization and embryo implantation. Hum Reprod Update. (2015) 22:dmv055–93. doi: 10.1093/humupd/dmv055, PMID: 26663221 PMC4755440

[44] Qu P Zhao Y Wang R Zhang Y Li L Fan J. Extracellular vesicles derived from donor oviduct fluid improved birth rates after embryo transfer in mice. Reprod Fertil Dev. (2019) 31:324–32. doi: 10.1071/RD18203, PMID: 30196804

[45] Orisaka M Jiang JY Orisaka S Kotsuji F Tsang BK. Growth differentiation factor 9 promotes rat Preantral follicle growth by up-regulating follicular androgen biosynthesis. Endocrinology. (2009) 150:2740–8. doi: 10.1210/en.2008-1536, PMID: 19213837

[46] Knight PG Glister C. TGF-β superfamily members and ovarian follicle development. Reproduction. (2006) 132:191–206. doi: 10.1530/rep.1.01074, PMID: 16885529

[47] Stocco C Telleria C Gibori G. The molecular control of Corpus luteum formation, function, and regression. Endocr Rev. (2007) 28:117–49. doi: 10.1210/er.2006-0022, PMID: 17077191

[48] Richards JS Russell DL Ochsner S Espey LL. Ovulation: new dimensions and new regulators of the inflammatory-like response. Annu Rev Physiol. (2002) 64:69–92. doi: 10.1146/annurev.physiol.64.081501.131029, PMID: 11826264

